# Main Aspects of Peripheral and Central Hearing System Involvement in Unexplained HIV-Related Hearing Complaints

**DOI:** 10.3389/fneur.2019.00845

**Published:** 2019-08-06

**Authors:** Marrigje Aagje de Jong, Ari Luder, Menachem Gross

**Affiliations:** Department of Otolaryngology—Head and Neck Surgery, Hadassah Hebrew-University Medical Center, Jerusalem, Israel

**Keywords:** inner ear, sensorineural hearing loss, HIV, audiometry, synaptopathy, ABR

## Abstract

**Objective:** Hearing abnormalities frequently occur in Human Immunodeficiency Virus (HIV) infected individuals. Both conductive and uni- or bilateral sensorineural hearing loss (SNHL) have been described along with other audiological and vestibular symptoms such as tinnitus, vertigo and balance disturbances. While frequent middle ear infections may explain impairment of peripheral hearing abilities, the exact etiology of cochlear, and central auditory processing deficits still remains unclear. Direct effects of HIV, opportunistic infections, ototoxic side effects of antiretroviral therapy (ART), and immunologic responses to the central nervous system involving the auditory pathway have been proposed. We aim to review the audiological profile in HIV infected adults related to the effects of HIV and HAART on the inner ear structures.

**Methods:** We present a review of the literature on cases of HIV related SNHL in adult patients and studies conducted to investigate audiometric changes in such patients. Data on presentation, diagnosis and pathophysiology were reviewed.

**Results:** Sensorineural hearing loss in the higher frequencies is a common form of hearing loss in HIV infected individuals throughout disease progression, along with decreased otoacoustic emission (OAE) responses, increased PTA hearing thresholds and prolonged latencies for auditory brainstem responses (ABR).

**Conclusion:** HIV affects all stages of auditory perception in a way similar to accelerated aging of the auditory system. And we postulate that synaptic loss may be the first step, followed by cochlear damage and central pathology as the virus remains present in all the structures of the auditory pathway causing local inflammation and degeneration. Evaluation of hearing function among all patients diagnosed with HIV infection seems to be an accepted approach; it should include OAE testing, pure tone and speech audiometry, speech-in-noise tests and ABR measurements.

## Introduction

The Human Immunodeficiency Virus (HIV) is a single-stranded RNA virus, which is converted to double-stranded DNA after infection of the host cell and integrates into its cellular DNA. HIV can infect a variety of cell types but preferentially infects neurons and immune cells, such as CD4^+^ T cells, macrophages, and microglial cells. Once integrated, the virus may become latent. This allows the virus and its host cell to avoid detection by the immune system. In its dormant state the virus can remain in the human body for several years without causing symptoms. With time, disease progresses and the neuro- and lymphotrophic virus causes functional impairment and depletion of T-cells resulting in immunosuppression, possible development of opportunistic infections and malignancies in multiple organ systems. With regard to its manifestations in the temporal bone, common symptoms include tinnitus, dizziness, chronic otitis media, and hearing impairment (1, 2).

It has been shown that HIV has affinity for the central nervous system (CNS) and in 20% of HIV infections the first symptom is a neurological manifestation (3). The CNS can act as anatomical reservoir for the virus resulting in cognitive deficits and other central pathologies including the auditory processing pathways (4).

Several studies have investigated hearing loss among HIV infected children and adults as well as those who developed Acquired Immune Deficiency Syndrome (AIDS) presenting a broad spectrum of auditory and otologic pathologies, ranging from middle ear infections to conductive, mixed and sensorineural hearing loss (SNHL), vestibular symptoms, and subcortical and cortical pathologies. It is our aim to provide an overview of those studies investigating hearing function among the adult HIV infected population. In addition to the hearing performance, we discuss the different hypotheses about the responsible mechanism(s) with a specific focus on inner ear and central neural pathology—an attempt to answer the question whether HIV affects mostly peripheral or central auditory processes.

## Methods

### Search Strategy and Selection

As a first step, a review of the literature was conducted using the databases of PubMed and the Cochrane Library on March, 2019. We searched for articles relating to patients with HIV infection (domain) with audiology testing (intervention) and subsequently, searched for articles limited to an adult population and published in the last 10 years. Articles were excluded if the main subject was not in relation to our domain in combination with determinant or in cases where the language was other than English. We screened references and related articles and conducted a general internet search to verify if all significant articles were included until day of admission of the manuscript.

We assessed and compared the hearing performances based on different audiology tests among patients with and without HIV infection in the selected studies. Due to heterogeneity in the study designs and the lack of standardized measurements, no meta-analysis was possible, and a critical conceptual review approach was chosen.

## Results

Based on our Pubmed and Cochrane search a total of nine articles were found and after subtracting the articles that did not meet the criteria of recent publication and/or adult population, only 4 remained. However, with articles found from references we selected a total of 13 relevant publications related to 11 different study populations from locations in Africa, Asia, South-America and the USA (2, 5–16).

In 6 articles the participants were asked to complete a questionnaire on self-reported hearing loss and/or otologic symptoms. Combined prevalence for each otovestibular complaint was as follows: aural fullness 30.5%, hearing loss 27.7%, tinnitus 20.9%, dizziness 24.9%, otalgia 15.8%, and aural pruritus 45% (2, 7, 9, 10, 13, 14). HIV infected patients receiving or not receiving antiretroviral therapy (ART) were not always analyzed separately. However, we noticed that for all otologic symptoms, except for aural fullness, the prevalence was slightly higher among those with ART and or longer disease process. Subjects receiving ART also reported greater difficulty understanding speech in noise (13).

### Evaluation of Peripheral Dysfunction of the Middle and Inner Ear

The pneumatization and function of the middle ear can be assessed by otoscopic evaluation and tympanometry—a readily easy test to perform, also in endemic areas that are most affected by HIV. A total of 7 studies described tympanometry in their study protocol (2, 4, 9–11, 13, 16, 17). It should be stated that often acoustic immittance testing was only performed to exclude middle ear pathology before continuing hearing screening. Van der Westhuizen et al. documented otoscopic abnormalities in 55% of HIV infected participants, and in 8% they found tympanic membrane perforation and/or otorrhea. The tympanogram was of type B in 34% of cases (9). Similar results were found by Matas et al. with 33.3% of HIV infected participants showing abnormal tympanograms, and 40.7% of HIV infected participants that were receiving ART (10).

Measurements of hearing at the level of the inner ear include: otoacoustic emission (OAE) tests, pure tone audiometry (PTA) with speech reception threshold (SRT) and word recognition scores (WRS) in quiet. Except for one study focusing on central auditory processing (2), all included studies described distortion product OAE measurement (DPOAE) and or PTA in their research method.

#### DPOAE Measurements

OAE tests represent cochlear function and were evaluated in 4 studies (7, 9, 12, 13). A slight decrease in normal DPOAE functioning throughout disease progression was seen (7, 9). In contrast Torre et al. found no significant difference in the number of no responses (NR) between HIV seropositive and HIV seronegative subjects (15). In two studies it is not clear whether subjects with abnormal tympanograms (33–34%) were excluded from DPOAE measures (9, 13). Details and outcomes of those studies are summarized in Table 1.

**Table 1:** Overview of distortion product otoacoustic emission (DPOAE) testing results—prevalence and conclusion.

**References**	**Year**	**Frequencies tested (Hz)**	**Definition abnormal or no response**	**HIV—group**	**HIV + group**	**ART/AIDS group**
Khosa-Shangase (7)	2011	750–1,000–2,000–3,000–4,000–6,000–8,000	<7 dB difference = abnormal response or a change of >10 dB in consecutive measurements (three sessions were done over 6 months' time)	–	All normal in 3 sessions (*n* = 16)	Abnormal responses for 6–8 kHZ in session 3, but no change >10 dB (*n* = 54)
Van der Westhuizen et al. (9)	2013	1,818–2,542–3,616–5,083–7,206	6–10 dB difference = abnormal response <6 dB difference = no response Abnormal DPOAE in 3 frequencies is considered an abnormal test result.	–	39–44% abnormal response (18–24% bilateral) (*n* = 112)	45% abnormal response (28% bilateral) (*n* = 78)
Torre et al. (12)	2014	2,000–3,000–4,000–6,000	<6 dB difference = no response or absolute level < −15 dB SPL no DPOAE response in 4 frequencies is considered an abnormal cochlear function	Number of NR's increased across frequencies (*n* = 220)	Number of NR's increased across frequencies. “No responses” were more common in low frequen-cies for women (*n* = 286)	–
Maro et al. (13)	2014	1,500–1,700–2,000–2,200–3,000–3,200–4,000–4,200–6,000–6,200–7,800–8,000	No definition	DPOAE's were reduced in the HIV infected population compared to healthy individuals (*n* = 302) There were no differences for DPOAE measurements between HIV infected subjects receiving ART (*n* = 319) or not receiving ART (*n* = 130)		

#### PTA Measurements

Audiometry was done for air conduction in all studies, but only in half of those, bone conduction was performed. The frequency range varied from 0.125 to 20 kHz. In most cases 0.25–8 kHz were evaluated. The definition of hearing loss based on the pure tone average was either defined as a pure tone average threshold >20 or >25 dB HL. Van der Westhuizen et al. investigated a limited range of frequencies for two different criteria of hearing loss (9). Table 2 presents an overview of the results regarding pure tone audiometry in the different studies. A total of 8 studies looked at variables such as gender, age, stage of disease, use of ART related to PTA hearing loss. The most prominent factor was increased age (5, 6, 11, 15, 16). Increased duration of disease, CD4^+^ cell count, and advanced stage of disease seemed to increase the risk of hearing loss, but findings were not consistent in all studies (5, 9, 11, 14, 16). While Torre et al. found no association between treatment variables and PTA (15), Matas et al. described an increased odds ratio for hearing loss in HIV infected individuals receiving ART compared to treatment naïve individuals (2). Other studies noticed that patients receiving ART seemed to have worse hearing at initiation of treatment with improvement when continuing ART (6, 14).

**Table 2:** Overview of Pure Tone Audiometry results—prevalence and type of hearing impairment.

**References**	**Year**	**Frequency range (kHz)**	**Definition HL (dBHL)**	**Prevalence HL HIV + (%)**	**Prevalence HL HIV + with AIDS /ART (%)**	**SNHL (%)**	**CHL (%)**	**Mixed HL (%)**	**HF SNHL (%)**	**Prevalence HL controls (%)**
Ongulo and Oburra (5)	2010	0.25–8	>25	33.5 (*n* = 194)	–	74	22	4	–	8.1 (*n* = 124)
Makau et al. (6)	2010	? − 8	>25	31 (*n* = 273)	28 (*n* = 271)	Most common	–	–	–	–
Khosa-Shangase (7)	2011	0.25–8	>25	–	10 (*n* = 150)	73	27	0	33	–
Mathews et al. (8)	2012	0.5–8	>20	54 (*n* = 30)	54 (*n* = 30)	37–81	0–12.5	6.25–12.5	–	–
Van der Westhuizen et al. (9)	2013	0.5–4	>15 >25	32 (*n* = 172) 12 (*n* = 172)	44 (*n* = 28) 18 (*n* = 28)	21–31 –	12–13 –	–	17	–
Matas et al. (10)	2014	0.25–20	>20	27.8–58.8 (*n* = 18)	48.1–73.9 (*n* = 27)	38.5	20–30.8	–	15.4–80	0 (*n* = 30)
Luque et al. (11)	2014	0.25–8	>25	16.5 (*n* = 127)	18.9 (*n* = 148)	100	–	–	–	11.6 (*n* = 137)
Maro et al. (13)	2014	0.5–8	>25 (>30/>20 for specific freq.)^*^	0.8 (*n* = 130)	1.3–2.5 (*n* = 319)	–	–	–	–	0–3.5 (*n* = 302)
Fokouo et al. (14)	2015	0.125–8	>20	27.2 (*n* = 90)	–	61.7	18.3	20	–	5.6 (*n* = 90)
Torre et al. (15, 16)	2015/16	0.25–8	No definition^**^	–	–	6.25–16	18.75–20	0–8	–	–
Matas et al. (10)	2018	0.25–20	No definition^***^	–	–	–	–	–	–	–

*HL, Hearing Loss; kHz, kilo Herz; dBHL, decibel Hearing Level; HIV +, HIV infected individuals with or without ART; AIDS, acquired immunodeficiency syndrome; ART, antiretroviral therapy; SNHL, sensorineural hearing loss; CHL, conductive hearing loss; HF SNHL, high frequency SNHL (among total SNHL); n, number of study participants for that group*.

* *PTA was not different for HIV infected individuals with or without ART, the HIV seronegative group had worse PTA outcomes*.

** *High and low frequency PTA were significant higher for HIV infected participants (n = 222) compared to healthy controls (n = 174)*.

*** *HIV infected participants (n = 41) had higher hearing thresholds in both conventional and high frequency audiometry when compared to the control group (n = 30)*.

#### Word Recognition Scores

As a measurement of retro-cochlear deficit, individuals can be asked to repeat word lists at comfortable hearing level. When done in quiet, the test can be too easy, not representing the daily listening environment leading to ceiling effects, even in those with hearing loss. Luque et al. and Torre et al. tested a 25-words list presented at 40–50 dB HL in quiet and found similar results (11, 15). The application of three-way ANOVA in the study of Luque et al. revealed higher WRS scores in HIV seronegative controls, and a significant difference compared to individuals with HIV infection at late stage disease (adjusted *P* = 0.03) (11). However, from a clinical perspective this difference seems inconsequential (16). There was also a significant effect of age on WRS regardless of HIV infection.

### Evaluation of Central Auditory Pathways

The Central Auditory Pathway is a complex system that can be tested objectively through auditory brainstem evoked responses (ABR), EcoG, middle, and late latency evoked responses (MLR, LLR), P300 wave measurement and other specific tests. With our search criteria we found two articles looking at central auditory processing through ABR or gap detection thresholds (2, 13). Matas et al. compared controls with HIV infected subjects and AIDS patients (2). ABR measurements were done for clicks (80 dBnHL, 19 clicks/s) to measure latencies and interpeak intervals. MLR measurements (70 dBnHL monoaural click, 9.9 clicks/s) were performed to determine contra- and ipsilateral amplitudes. ABR latencies were prolonged for the HIV seropositive subjects and AIDS patients compared to controls, as well as the I-V interpeak latency (4.39 and 4.33 vs. 4.05 ms). MLR in HIV infected subjects showed prolonged latencies and decreased amplitudes when the electrode was placed at C3 compared to seronegative controls. The difference was most obvious for AIDS patients. P300 is an electric wave in the electric encephalogram that occurs at 300 ms after stimulus and is often measured at the posterior aspect of the skull. Matas et al. found also here significantly prolonged latencies for HIV infected and AIDS diseased participants (2). The Gap detection threshold was evaluated in HIV infected subjects with or without ART (13). Measured at 65 dB, a slight increase in gap detection threshold was found among HIV infected participants compared to seronegative controls.

As some studies suggest, people with HIV can suffer from hearing complaints but show no objective abnormality in the audiometry (11, 18). ABR measurements are not clearly affected in all HIV patients with hearing complaints either. This makes it difficult to separate clearly between peripheral and central origins in HIV infected subjects with hearing complaints. A more recent hypothesis for a hidden form of hearing loss is synaptopathy. However, we found no study focusing on synaptopathy specifically in the HIV infected population.

## Discussion

It is known that HIV infected individuals who develop immunosuppression are more susceptible to several inflammatory and infectious processes affecting the temporal bone (1). This can result in conductive hearing loss, sensorineural damage or a mixed loss. However, auditory and vestibular complaints such as hearing loss and tinnitus are frequently mentioned in self-reports by adults, already in the early stages of symptomatic HIV infection (2, 6, 7, 9, 10, 14, 15). In particular, difficulty in understanding speech in noise is reported and data suggest a progression throughout disease stages (4). Our review summarizes the results from the most recent studies conducted to evaluate the middle ear, inner ear and central auditory pathways in adult patients with HIV infection and/or AIDS disease. Can we answer our question whether HIV impairs hearing function at a more peripheral or central etiology?

As stated before, it is not easy to discern, based on previous works, whether the main problem is peripheral or central. For simple reasons such as a wide variety in patients selected, differences in accessibility to auditory screening and HIV treatment, variable methods for hearing evaluation and the new developments and early initiation of ART possibly affecting their outcomes. Although no consistent pattern has been established, DPOAE's showed abnormal responses mainly in higher frequencies and decreased over time with disease progression (7). Decreased OAE's often precede alterations in normal audiograms and represent early cochlear damage, as occurs after noise exposure or ototoxic treatment (12, 17, 19). A variety of studies have hypothesized that some drugs used for ART (in particular the first line antiretroviral drugs known as NRTI's) cause such ototoxic damage or increase sensitivity to noise (20–23).

In 1995 Lewis described the phenomenon of ART related ototoxicity through damaged mitochondrial DNA causing oxidative stress leading to accelerated changes on the cochlea or central auditory system similar to those described in aging humans (24). Increasing evidence now suggests that HIV infected individuals experience similar immunologic changes as seen in elderly persons with accelerated aging and related co-morbidities. It is thought that immune activation and inflammation translates into a general decline of the immune system resulting in those changes (25). This phenomenon is called immunosenescence or aging of the immune system and can very well play a role in HIV related hearing loss through damaged auditory pathways by HIV itself rather than through ART.

From the recent literature we selected, it is found that HIV individuals do not show poorer DPOAE test results after ART is initiated. While age was related to decreased responses, all variables related to HIV status (HIV viral load, CD4^+^ cell count) and ART did not show significant correlation (7, 12, 13). A significant ototoxic effect from ART based on OAE testing is therefore unlikely.

Secondly, the presence of HIV in cochlear structures has been described by Pappas et al. (26). They found extracellular viral-like particles with morphologic characteristics of HIV-1 on the tectorial membrane in three cases. Numerous viral-like particles appearing essentially similar to identified HIV-1 particles in infected lymphocyte cultures were found within the cytoplasm of the connective tissue cells. These findings provide an insight into the cochlear pathogenesis of viral-induced hearing loss and vestibular impairment in HIV infected patients. Through possible persistent inflammation and immune activation.

When looking at PTA results, there is an undoubtedly higher prevalence of hearing loss among HIV infected individuals—mostly of the sensorineural type and in the higher frequencies. Although PTA changes were often documented as a mild to moderate loss, there is a trend toward progression with longer disease (5, 9, 14–16). When CD4+ cell counts drop to below 200 cells/μL immunological AIDS develops and is often indication to start ART. Some studies found an initial increase in PTA thresholds at this stage, but when ART was continued the prevalence of hearing loss decreased (14, 15). A possible explanation is the efficacy of ART on the immune system and a viral suppression leading to hearing improvement. However, contradictive results were found regarding the benefit of ART on hearing performance. Matas et al. and Van der Westhuizen et al. found a higher prevalence of SNHL among those receiving ART for advanced disease (9, 10). To differentiate between the effect from ART and from advanced disease is however difficult. It is known that second-line regimens are composed of newer and safer compounds than those used in first-line regimens (14). However, penetration of ART to the CNS is less optimal and can lead to incomplete recovery of the immune system. Even with good systemic control, HIV can persist in the CNS putting infected subjects at risk for cognitive impairment which continues to be prevalent in particular among those over 50 years old (11).

A variety of studies have hypothesized that HIV, while present in the CNS, has its direct effect on the vestibular and cochlear system as well as more central auditory processing pathways (13, 27, 28). As demonstrated by Matas et al. and Maro et al. MLR latencies and gap detection thresholds were prolonged as a measurement for central auditory processing (2, 13). Matas et al. also found prolonged wave I, III, and V latencies during ABR measurements similar to previous works (2, 29). Wave I represents the peripheral part of the auditory nerve at the spiral ganglion cells that connect the hair cells in the cochlea with the next neural processing unit, the cochlear nucleus, where wave III is generated (30). Wave III and V are delayed when more central conduction pathways are affected. Although the I-V interpeak latency was the only significant interpeak delay, the results by Matas et al. show that interpeak time I-III was more prolonged compared to interpeak III-V (2). This may correspond to the action potential conduction time from the hair cell to the cochlear nucleus and appears to be affected in HIV infected individuals from the study (30). Previously it was thought that hair cells were the most vulnerable elements to cochlear injury and aging. But recent work shows that loss of synapses and nerve fibers (known as synaptopathy) occur even before hair cell damage (18). The number of surviving cochlear synapses is represented by the amplitude of wave I in ABR testing. In this review we did not find recent data on the amplitudes in ABR among HIV infected adults. But with complaints of difficulty in understanding speech in noise, wave I abnormalities in ABR and a possible explanation of accelerated aging in HIV disease, it can be useful to conduct further research in this field among the HIV infected population. Since cochlear synaptic loss does not present itself in cochlear measurements such as OAE's and PTA (hidden hearing loss), it will be a challenging task.

In summary, auditory tests included in the present review showed overall decreased DPOAE's with disease progression, SNHL in the higher tones during PTA related to progression of disease and in particular to older age. Together with poorer word recognition scores in quiet, this is suggestive for cochlear damage. But central auditory measurements such as gap detection thresholds, ABR and MLR tests and the P300 also confirmed CNS involvement among HIV seropositive subjects. The affected processing of sound in HIV infected subjects is therefore not merely a peripheral or a central pathology. HIV affects all stages of auditory perception in a way similar to accelerated aging of the auditory system. And we postulate that synaptic loss may be involved, however further study is needed to evaluate this possible explanation. As well as cochlear damage and central pathology as the virus remains present in all the structures causing local inflammation and degeneration. Health care providers should be aware of the increased risk of hearing loss among HIV infected persons that are under their treatment. Early detection and the use of several auditory measurements is recommended. It should include OAE testing, pure tone and speech audiometry, speech in noise tests and ABR measurements.

## Author Contributions

MdJ, AL, and MG all contributed to the writing of this article.

### Conflict of Interest Statement

The authors declare that the research was conducted in the absence of any commercial or financial relationships that could be construed as a potential conflict of interest.
